# Knowledge, skills and attitudes of hospital pharmacists in the use of information technology and electronic tools to support clinical practice: A Brazilian survey

**DOI:** 10.1371/journal.pone.0189918

**Published:** 2017-12-22

**Authors:** Eugenie Desirèe Rabelo Néri, Assuero Silva Meira, Hemerson Bruno da Silva Vasconcelos, David John Woods, Marta Maria de França Fonteles

**Affiliations:** 1 Surveillance and Patient Safety Sector, Assis Chateaubriand Maternity School, Federal University of Ceará, Fortaleza, Ceará, Brazil; 2 Postgraduate Program in Pharmaceutical Sciences, Faculty of Pharmacy, Dentistry and Nursing, Federal University of Ceará, Fortaleza, Ceará, Brazil; 3 Planning Sector, Assis Chateaubriand Maternity School, Federal University of Ceará, Fortaleza, Ceará, Brazil; 4 School of Pharmacy, Otago University, Dunedin, New Zealand; University of Tennessee Health Science Center, UNITED STATES

## Abstract

This study aimed to identify the knowledge, skills and attitudes of Brazilian hospital pharmacists in the use of information technology and electronic tools to support clinical practice. Methods: A questionnaire was sent by email to clinical pharmacists working public and private hospitals in Brazil. The instrument was validated using the method of Polit and Beck to determine the content validity index. Data (n = 348) were analyzed using descriptive statistics, Pearson's Chi-square test and Gamma correlation tests. Results: Pharmacists had 1–4 electronic devices for personal use, mainly smartphones (84.8%; n = 295) and laptops (81.6%; n = 284). At work, pharmacists had access to a computer (89.4%; n = 311), mostly connected to the internet (83.9%; n = 292). They felt competent (very capable/capable) searching for a web page/web site on a specific subject (100%; n = 348), downloading files (99.7%; n = 347), using spreadsheets (90.2%; n = 314), searching using MeSH terms in PubMed (97.4%; n = 339) and general searching for articles in bibliographic databases (such as Medline/PubMed: 93.4%; n = 325). Pharmacists did not feel competent in using statistical analysis software (somewhat capable/incapable: 78.4%; n = 273). Most pharmacists reported that they had not received formal education to perform most of these actions except searching using MeSH terms. Access to bibliographic databases was available in Brazilian hospitals, however, most pharmacists (78.7%; n = 274) reported daily use of a non-specific search engine such as Google. This result may reflect the lack of formal knowledge and training in the use of bibliographic databases and difficulty with the English language. The need to expand knowledge about information search tools was recognized by most pharmacists in clinical practice in Brazil, especially those with less time dedicated exclusively to clinical activity (Chi-square, p = 0.006). Conclusion: These results will assist in defining minimal competencies for the training of pharmacists in the field of information technology to support clinical practice. Knowledge and skill gaps are evident in the use of bibliographic databases, spreadsheets and statistical tools.

## Introduction

The use of information technology and automation to support pharmacy practice dates from the 1970s [1, 2]. From 1999, large investments were made in this area aiming to reduce medication errors and increase patient safety [3, 4]. To respond to the change, pharmacists require dedicated training in information technology and electronic tools.

In Brazil, the national curricular guidelines for undergraduate pharmacy education, published in 2002, define the principles, fundamentals, conditions and procedures for the training of pharmacists in this country. These curricular guidelines present the need to master the use of information technology to exercise competency in health care decision making, communication, leadership, management, and continuing education [5]. In our view, little attention has been given to training in the knowledge and skills in information technology and electronic tools in the undergraduate pharmacy program in Brazil. Similarly, there has been little attention to training programs and continuing education in these disciplines for practicing pharmacists.

In Brazil, the practice of clinical pharmacy is increasingly dependent on appropriate knowledge and practical skills in the use of information technology. The use of these tools facilitates professional communication, including the use of electronic medical records and the maintenance of accurate medication profiles. They are also used for pharmacotherapeutic follow-up, requesting and documenting laboratory and diagnostic tests and for the reporting of adverse drug reactions. The use of information technology is recognized as an essential requirement for the development of clinical pharmacy in Brazil [6].

Information technology is also an essential element in the education of health professionals [7, 8] and is widely used in pharmaceutical practice [9–11], especially in searching for information [8, 9, 11]. Consequently, lack of training in this area could have a negative impact on health care. Identification of training needs and knowledge gaps are thus important strategies for the improvement of professional practice.

The objective of this study was to determine the profile of Brazilian hospital pharmacists active in clinical practice with respect to basic skills in the use of information technology and electronic tools that support clinical practice. This would help to identify opportunities for professional development in this area to respond to the increasing use of information technology in Brazilian hospitals.

## Material and methods

### Study setting and population

This is an exploratory study conducted in Brazil, with clinical pharmacists, who work in public and private hospitals (n = 1373).

### Sampling strategy

The minimum sample size was calculated on the basis of the First Brazilian Census of Hospital Pharmacy, carried out by the Federal Pharmacy Council that indicates the number of hospital pharmacists in Brazil [12]. Considering a 95% confidence interval (p<0.05) and that 25% of hospital pharmacists have clinical tasks (n = 1373), the minimum sample size for the results to be representative was defined (n = 274), and stratified by state, according to census data.

### Study tool

A questionnaire totaling 26 questions was developed in Portuguese, with open and closed questions (S1 Appendix). The questions included in the questionnaire were adapted and developed from similar questionnaires [7, 8, 10, 13, 14] and the clinical activities described in the Brazilian regulation of clinical pharmacy [6]. Our survey included open ended questions (questions 1 and 26) and closed questions (binary: questions 2, 8 and 24; Likert scale ratings: questions 7,11,12,14 and 25; and multiple-choice: questions 3–6, 9, 10, 13, 15–23) [15].

### Validity and reliability of the study tool

The questionnaire used in the present study was previously validated by a committee [16] composed of 10 hospital pharmacists with clinical experience (three second-year resident pharmacists, five pharmacists with different levels of experience, and two professors, one with a master’s degree and the other with a doctorate degree).

The specialists were invited to evaluate the relevance of each question and the clarity of the content of each item [17, 18, 19]. The criterion of relevance considered the importance and appropriateness of the question to achieve the proposed objectives (relevance scale: 1-irrelevant, 2-somewhat relevant, 3-relevant and 4-very relevant) [20], and if all necessary dimensions of the objective were included. In relation to clarity, the editing of the items was evaluated such that the concept expected to be measured was fully understandable and adequately expressed (clarity scale: 1-not clear, 2-somewhat clear, 3-clear and 4-very clear) [18].

For the validation of the questionnaire, we determined the index of content validity (CVI). This measures the proportion or percentage of evaluators who are in agreement on certain aspects of the instrument, and of its items for each question as well as for the questionnaire as a whole (formula used: CVI = number of responses categorized as 3 or 4 for clarity or relevance divided by the total number of responses). Polit and Beck recommend a CVI of 0.9 of higher; questions with a CVI of less than 0.9 were adjusted and re-evaluated for incorporation into to the final questionnaire [20]. The details of the validation study are not included in the results of this study.

### Ethical approval

The study was approved by the Ethics in Research Committee, Federal University of Ceará, CAAE: 44308815.7.0000.5054

### Sample recruitment and data collection

The subjects of this survey were recruited from Brazilian hospital pharmacists that work in clinical tasks (n = 1373) and were registered by the Regional Pharmacy Boards of Brazil and members of the Brazilian Society of Hospital Pharmacy and Health Services-SBRAFH. The questionnaire, presented on the Google docs platform (Attachment 1), was sent by e-mail to participants. Each pharmacist provided informed consent and filled the questionnaire without intervention of the researcher. The response was linked to a Google account, allowing only one response per participant. The respondent’s email address was not recorded on the response form but was used to disallow a second response. Data collection occurred from July 27 to September 27, 2015.

The research was widely disseminated in groups of clinical pharmacists on Facebook and on the website https://farmaceuticoclinico.com.br/, by a link that presented the research objective, the composition of the questionnaire, and reinforced the importance of the participation of invited pharmacists in the study.

The administration of the questionnaire was carried out after validation. Responses were recorded by all pharmacists who provided consent and who also indicated involvement in clinical activities in hospital pharmacy. Responses were excluded from the study if the respondent indicated that he/she did not have clinical duties or was involved in clinical activities exclusively in non-hospital units. The exclusions (n = 9) did not alter the original estimate of required sample size.

### Data analysis

Data were analyzed using descriptive statistics. Pearson's Chi-square test was used for categorical variables and the Gamma correlation test was used to analyze the probability distribution. In this article we present the analysis of the results of questions 1–8; 10–13 and 15–17 from the questionnaire. The results obtained in the other questions will be presented later in other publications.

## Results

### Response rate

The questionnaire was filled by 357 hospital pharmacists. After analysis of the responses, nine questionnaires were excluded from the study because the respondent indicated that they did not have clinical duties or was involved in clinical activities exclusively in non-hospital units (S2 Appendix). In this study, the response rate was 348/1373 (25.3%).

### Background characteristics of the samples

Responses were obtained from all states of Brazil and the Federal District. The profile of participants is presented in Table 1

**Table 1:** Profile of study respondents.

Variable		n	%
Sex (n = 348)	Male	106	30.5
	Female	242	69.5
Age group	22 to 25 years	42	12.1
	26 to 30 years	101	29.0
	31 to 40 years	131	37.6
	41 to 50 years	56	16.1
	Over 50 years	18	5.2
Time in clinical pharmacy practice	< 1 year	103	29.6
	1–5 years	185	53.2
	6–10 years	36	10.3
	11–20 years	18	5.2
	>20 years	6	1.7
Number of hours/week dedicated exclusively to clinical pharmacy	<2 h	60	17.2
	2–6 h	99	28.5
	7–12 h	56	16.1
	13–24 h	46	13.2
	25–40 h	87	25.0
Type of establishment	Not indicated	1	0.3
	Private	87	25.0
	Public	260	74.7
Place of establishment	Not indicated	22	6.3
	Capital	266	76.5
	Interior	60	17.2
Size of establishment	Not indicated	24	6.9
	Up to 50 beds	21	6.0
	Between 51 and 150 beds	95	27.3
	>150 beds	208	59.8

With respect to the number of hours dedicated exclusively to clinical pharmacy practice in a hospital environment, the results did not show statistically significant differences between public and private hospitals (Chi-square test: p = 0.417), between hospitals in the capital (the main city of a state) and in interior (the cities that are not the main city) (Chi-square test: p = 0.286), or between the Northeast, Southeast and South regions (Chi-square test: p = 0.079). However, a positive association was found between the number of beds in the health care establishment and increased time dedicated by pharmacists exclusively to clinical pharmacy (gamma correlation test: p = 0.032).

Pharmacists had at least one and at most four electronic devices (mobile or desktop) for personal use (their own equipment used for leisure, study and work). These were laptops (81.6%; n = 284), smartphones (84.8%; n = 295), desktop computers (46.8%; n = 163) and tablets (37.4%; n = 130). In the workplace 89.4% (n = 311) had a computer and in most cases with access to the internet (83.9%; n = 292), facilitating access to information. Most pharmacists (97.1%; n = 338) believed that having access to the internet helped in clinical practice. Most respondents used a computer mainly to search for clinical information (92.5%; n = 322), exchange information by e-mail or intranet (88.8%; n = 309), and to control and dispense medicines (70.7%; n = 246).

When questioned about time spent per week using devices connected to the internet (for all activities including leisure, work, and study), 59.5% (n = 207) reported being connected for longer than 11h per week and the preferred internet browser was Google Chrome (74.1%; n = 258). There was no significant difference in time of access to the internet between sexes (Chi-square test: p = 0.081), between age groups of the pharmacists (gamma correlation test: p = 0.726), and between respondents from the Northeast, Southeast and Southern regions of Brazil (Chi-square test: p = 0.078).

### Evaluation of knowledge, skills and attitudes in use of the internet and software

With respect to basic skills in the use of the internet and software (Table 2) most of the pharmacists evaluated themselves as being “very capable” to search for a page on the internet (95.4%; n = 332), as well as downloading a file from a site on the internet (87.1%; n = 303). However, 69.5% (n = 242) of the pharmacists reported not having received formal education in using the internet.

**Table 2:** Evaluation of skills and formal education of Brazilian clinical pharmacists in use of the internet and software.

	Evaluation				Formal education ^a^	
Skills (n = 348)	Very capable % (*n*)	Capable % (*n*)	Somewhat capable % (*n*)	Incapable % (*n*)	Yes % (*n*)	No % (*n*)
**Internet: search for a web page/web site on a specific subject** ^b^	95.4 (332)	4.6 (16)	0.0 (0)	0.0 (0)	30.5(106)	69.5 (242)
**Internet: Download a file from a site**	87.1 (303)	12.6 (44)	0.3 (1)	0.00 (0)		
**Use a spreadsheet (e.g., Excel)**	41.7 (145)	48.5 (169)	9.8 (34)	0.0 (0)	44.8 (156)	55.2 (192)
**Use statistical analysis software Epi Info**	5.1 (18)	21.3 (74)	46.0 (160)	27.6 (96)	13.5 (47)	86.5 (301)
**Use statistical analysis software SPSS**	4.3 (15)	17.3 (60)	42.8 (149)	35.6 (124)	11.8 (41)	88.2 (307)
**Search using MeSH terms**	60.3 (210)	37.1 (129)	2.6 (9)	0.00 (0)	52.6 (183)	47.4 (165)
**Search for articles in databases (e.g., PubMed, Medline, BVS/Bireme)**	46.3 (161)	47.1 (164)	5.7 (20)	0.9 (3)	56.3 (196)	43.7 (152)
**Overall evaluation of skills (n = 1948)**	47.5 (925)	21.9 (427)	19.2 (373)	11.4 (223)	34.9 (729)	65.1 (1359)

SPSS- Statistical Package for the Social Sciences; BVS-Virtual Library in Health (http://brasil.bvs.br/).

^a^ Formal education is a program that is officially recognized, offered in schools as courses with grades, degrees, programs, curricula and diplomas [20].

In relation to skills in the use of spreadsheets, the percentage of pharmacists who considered themselves “very capable” was less than that of “capable,” possibly reflecting lack of formal education in the subject, reported by 55.2% (n = 192) of the pharmacists. The use of spreadsheets is common among Brazilian pharmacists, especially in hospitals with a low degree of computerization, and knowledge about them is important to allow appropriate use.

Knowledge of the use of statistical analysis tools (examples: Epi Info and SPSS) can assist the pharmacist in analyzing data generated in clinical practice. In this regard, most pharmacists responded as being “somewhat capable” or “incapable” in the use of this software. This was probably due low rates of formal education in this discipline (Epi Info: 13.5%; n = 47 and SPSS 11.8%; n = 41) (Table 2). Conversely, most pharmacists responded as being "very capable" or "capable" in searching for web pages, downloading files from the internet, searching using MeSH terms, searching for articles in databases and using spreadsheets. However, for most of these activities, most pharmacists did not receive formal education (Table 2).

In searching for information, Google, Micromedex, Up to Date and Medscape were the most frequently used resources accessed daily. PubMed, BVS/Bireme, Google Scholar were also accessed regularly. MD Consult and Scopus were used rarely or never used by pharmacists to support clinical practice and were also associated the lowest levels of self-reported skill. The highest level of skill was reported with Google, Google Scholar and Micromedex. (Table 3).

**Table 3:** Pharmacists’ use of evidence search tools.

	Frequency of use ^a^ %; (n)						Skill in use %; (n)			
Evidence search tools	Daily	Weekly	2 or 3 a month	Once a month	Rarely ^b^	Never used	Very capable	Capable	Somewhat capable	Incapable
**Google**	78.7 (274)	12.6 (44)	2.6 (9)	0.6 (2)	5.5 (19)	0.0 (0)	84.2 (293)	15.5 (54)	0.3 (1)	0.0 (0)
**Google Scholar**	22.7 (79)	27.9 (97)	14.7 (51)	5.2 (18)	20.9 (73)	8.6 (30)	60.1 (209)	26.1 (91)	7.5 (26)	6.3 (22)
**BVS/Bireme**	8.9 (31)	21.6 (75)	20.1 (70)	10.3 (36)	25.6 (89)	13.5 (47)	18.4 (64)	43.4 (151)	23.8 (83)	14.4 (50)
**PubMed**	18.4 (64)	30.4 (106)	21.0 (73)	8.6 (30)	14.7 (51)	6.9 (24)	33.0 (115)	40.0 (139)	19.0 (66)	8.0 (28)
**MD Consult**	1.7 (6)	6.6 (23)	5.5 (19)	3.4 (12)	14.1 (49)	68.7 (239)	3.2 (11)	12.1 (42)	19.8 (69)	64.9 (226)
**UpToDate**	30.7 (107)	14.1 (49)	7.8 (27)	3.4 (12)	16.7 (58)	27.3 (95)	31.0 (108)	26.7 (93)	16.1 (56)	26.2 (91)
**Scopus**	3.4 (12)	10.9 (38)	7.5 (26)	2.6 (9)	18.7 (65)	56.9 (198)	8.6 (30)	16.4 (57)	19.3 (67)	55.7 (194)
**Medscape**	21.8 (76)	21.0 (73)	12.7 (44)	6.9 (24)	18.1 (63)	19.5 (68)	28.4 (99)	28.7 (100)	21.3 (74)	21.6 (75)
**Micromedex**	50.3 (175)	16.7 (58)	8.0 (28)	5.2 (18)	10.1 (35)	9.7 (34)	48.0 (167)	29.3 (102)	13.2 (46)	9.5 (33)

^**a**^ Sample size, n = 348.

^b^ Less than once a month.

Most of the hospital pharmacists (73.8% (n = 257) reported having access to UpToDate, MD Consult or others to search for evidence to support clinical practice, however, they used daily Google as their main search tool (Table 3).

Finally, 63.2% (n = 220) of pharmacists with clinical duties believed that they needed to expand their knowledge of the use of evidence search tools to improve their work. This need is perceived to be greater by the pharmacists that have less time in clinical practice (Gamma correlation test: p = 0,001) (Table 4).

**Table 4:** Perception of the need to broaden knowledge of use of evidence search tools.

Time in clinical pharmacy	I need to expand my knowledge of the use of search tools for evidence to improve my work. %; (n)		
	Disagree and indifferent ^a^	Partially agree	Totally agree
< 1 year ^b^	11.7 (12)	12.6 (13)	75.7 (78)
1 to 5 years ^b^	10.3 (19)	29.2 (54)	60.5 (112)
6 to 10 years ^b^	13.9 (5)	27.8 (10)	58.3 (21)
11 to 20 years ^b^	27.8 (5)	38.9 (7)	33.3 (6)
>20 years ^b^	33.3 (2)	16.7 (1)	50.0 (3)
Total	12.4 (43)	24.4 (85)	63.2 (220)

^a^ The categories “Totally disagree,” “Partially disagree” and “Do not agree or disagree” (indifferent) were combined due to the low number of responses in the last two categories.

^b^ Gamma correlation test: p = 0.001.

## Discussion

### Main results

By conducting this survey we found that hospital pharmacists with clinical tasks had several electronic devices for personal use, mainly smartphones and laptop computers and were connected to the internet for more than 11 hours per week. At work, most pharmacists also had access to a computer connected to the internet.

Pharmacists believed that access to the internet facilitates access to information and assists in clinical practice. They also felt competent in performing basic activities in the use of the internet, such as searching for a web page, downloading files, using spreadsheets, searching using MeSH terms and searching for articles in databases, even though most reported not having received formal education for such activities. Regarding the use of statistical analysis software (Epi Info and SPSS), most pharmacists declared that they felt "not capable" or "incapable" of using them, and that they had not received formal instruction to do so. SPSS and Epi Info are the tools more frequently cited by Brazilian hospital pharmacists in articles published in the Brazilian Society of Hospital Pharmacy and Health Services-SBRAFH journal.

Access to databases (such as MD Consult and UpToDate) was available to most pharmacists participating in the survey; however, most reported much greater use of Google, a non-specific search engine. Most hospital pharmacists in clinical practice in Brazil recognized the need to expand their knowledge about information search tools to improve performance in clinical tasks, especially those with less time dedicated exclusively to clinical activity.

### Interpretation

This study demonstrated that Brazilian hospital pharmacists performing clinical activities regularly access the internet and use electronic tools to support their clinical practice. However, the full potential for use of these tools is not being realized due to significant knowledge gaps and lack of training on this subject in the undergraduate program and during professional education [10, 13, 21, 22]. Information technology is widely used by hospital pharmacists and many felt competent in the use of these tools and resources but, measured competence and the impact of information technology on quality of practice and patient outcomes have not been assessed by formal training and evaluation. Information management in hospitals is an essential component of patient care. However, meaningful use of this information may be hampered because of the exponential increase in the quantity and variable quality of available information on the internet [8, 23, 24] and the language in which information is published [21]. In view of this, it is important that professionals know and adopt criteria to properly select and evaluate the information obtained. The use of pre-appraised, specialized search databases are preferred instead of broad non-specific search engines such as Google, in which the information offered is prone to lack scientific support. The lack of training in the use of databases based on scientific evidence makes it difficult for the user to retrieve relevant information. Poor quality information hinders the application of evidence-based practice [25].

The Brazilian Society of Hospital Pharmacy and Health Services-SBRAFH, states in its minimum standards a recommendation that hospital pharmacists “should participate in activities overseeing the use of information technologies and should use evidence-based information in their practice” [26]. However, there is no current initiative to implement an educational program or minimum standards of knowledge for hospital pharmacists to respond to the increasing use of information technology in hospitals [11].

### Comparison with other results

In this study, the obtained response rate was similar to the expected for electronic questionnaires (25 to 26%) [27], and our results are consistent with other studies which evaluated the knowledge and skills of pharmacists in the use of electronic tools [10, 14, 21, 22].

The use of Google as the main information search tool was also observed in a study conducted in among pharmacists in Greece [22] and in students in the United Arab Emirates [21]. These results show the importance of strengthening pharmacists’ training in the use of evidence-based databases during undergraduate programs and professional life to increase appropriate use in practice.

We also believe that in our study, similar to observations from the study of Greek pharmacists [22], that the choice of using Google as the main search tool may have been influenced by difficulty with English language used in the main databases.

### Limitations

Our study had limitations that could have influenced some of the results. The index of content validity used is subjective and this evaluation could have been supplemented by application of other psychometric measures. Also, the fact that the questionnaire was made available electronically and by means of Google Docs could have produced bias in selection, favoring professionals who use the internet more widely and those who have Google e-mail.

## Conclusions

To our knowledge, this is the first Brazilian investigation of the use of information technology and electronic tools to support the clinical practice of pharmacists. Therefore, we anticipate that these results will be used to justify training programs to address current gaps in knowledge and skills in the use of IT, software and evidence-based databases. This in turn should lead to improvement in clinical practice.

For the future, it is strategic to investigate how the pharmacist is being trained to make the use of the information generated (big data); to obtain health information from everyday objects (internet of things) and to improve communication. In this sense, other research should be conducted in Brazil.

## Supporting information

S1 Appendix Questionnaire on knowledge, skills and attitudes in the use of information technology (internet and software) to record clinical practice, in the daily routine of Brazilian hospital pharmacists. (DOCX)

S2 Appendix Data file of the study (n = 348). (XLSX)
