# Supplementary material for: Knowledge, skills and attitudes of hospital pharmacists in the use of information technology and electronic tools to support clinical practice: A Brazilian survey
Source: PLoS One. 2017 Dec 22;12(12):e0189918. doi: 10.1371/journal.pone.0189918 (PMC5741238; doi:10.1371/journal.pone.0189918)
Supplement: S1 Appendix — (DOCX) [file pone.0189918.s001.docx]

S1 Appendix. Questionnaire on knowledge, skills and attitudes in the use of information technology (internet and software) to record clinical practice, in the daily routine of Brazilian hospital pharmacists.

**1. What is the Brazilian state where you work? _______________**

**2. What is your gender?**  Male Female

**3. What is your age group?**

<22 years

22 to 25 years

26 to 30 years

31 to 40 years

41 to 50 years

Over 50 years

**4. Devices you own for personal use:**

Which devices do you personally own and use for leisure, study or work.

Desktop computer Smartphone

Laptop Tablet None of these

**5. Which web browser do you use most often for internet access?**

An Internet browser is a program which allows you to access the Internet, watch videos, listen to music, play and interact with virtual documents.

 Google Chrome Internet Explorer

Mozilla Firefox Other_________________

**6. How many hours per week do you estimate that you use a desktop computer, laptop, tablet and smartphone with internet access?**

The response to this item should include total Internet usage time for leisure, work, study, etc.

 < 1h  11 - 15h

 1 - 5h  >15h

 6 - 10h

**7. Your internet and software skills; assess your ability to:**

|  | Very capable | Capable | Somewhat incapable | Incapable |
| --- | --- | --- | --- | --- |
| Search for a web page/web site on a specific subject |  |  |  |  |
| Download a file from an internet site |  |  |  |  |
| Search MeSH terms |  |  |  |  |
| Search articles in databases (e.g: PubMed^a^, Medline^b^, BVS/Bireme^c^, etc) |  |  |  |  |
| Perform efficient management of information for application in clinical practice ^d^ |  |  |  |  |
| Use spreadsheet (e.g.:Excel) |  |  |  |  |
| Use Statistical Analysis Software: Epi Info^e^ |  |  |  |  |
| Use Statistical Analysis Software: SPSS^f^ |  |  |  |  |

^a^ PubMed: It is a service of the U.S. National Library of Medicine that provides access to a free database of citations, abstracts, and entire articles provided by MEDLINE (https://www.ncbi.nlm.nih.gov/pubmed)

^b^ Medline: Medical Literature Analysis and Retrieval System Online. It is a database of the international medical and biomedical literature, produced by the National Library of Medicine, USA - NLM, which contains bibliographical references and abstracts of journals published in the United States and in other 70 countries. (NB PubMed is the same as Medline but they are available via different platforms)

^c^ BVS/Bireme: It is the Biblioteca Virtual em Saúde/ Virtual Library in Health, developed under the coordination of the Latin American Center for Health Sciences Information (BIREME). It is a network of on-line information sources for the distribution of scientific and technical knowledge in health (http://bvsalud.org).

^d^ Efficient information management is understood as the process in which information forms the basis of the decision-making process, enabling planning, execution and evaluation of the actions to be developed. From it, the professional modifies procedures, evaluates alternatives and elaborates solutions.

^e^ Epi Info is a public domain suite of interoperable software tools designed for the global community of public health practitioners and researchers, by Center for Disease Control and Prevention-CDC/USA.

^f^ SPSS is a IBM software used for statistical analysis of data.

**8. Have you received formal education ^g^ to use / perform:**

|  | Yes | No |
| --- | --- | --- |
| Internet |  |  |
| Text editor (e. g.: Word) |  |  |
| Spreadsheet (e. g. : Excel) |  |  |
| Epi Info |  |  |
| SPSS |  |  |
| Search MeSH terms |  |  |
| Search in database (e.g.: BVS/Bireme, PubMed) |  |  |

^g^ Formal education is a program that is officially recognized, offered in schools in courses with levels, degrees, programs, curricula and diplomas.

**9. In clinical practice, you use the internet to:**

Search for mechanism of action of medicines

Search for drug doses

Search for drug-drug interactions

Search for drug-food interactions

Search for adverse drug reactions

Search for dosage forms

Learn about clinical pharmacy methods

Look for information on how to document clinical pharmacy activities

Information about disease pathology

Search for drug incompatibility

Do conversion of commercial name into generic and vice versa

Others ___________

**10. How frequently do you search for information in clinical practice?**

|  | Daily | Weekly | 2 to 3 times a month | Once a month | Rarely (less than once a month) | Never used |
| --- | --- | --- | --- | --- | --- | --- |
| Google ^h^ |  |  |  |  |  |  |
| Google scholar ^i^ |  |  |  |  |  |  |
| Bireme |  |  |  |  |  |  |
| PubMed |  |  |  |  |  |  |
| Scopus ^j^ |  |  |  |  |  |  |
| UpToDate ^k^ |  |  |  |  |  |  |
| MD Consult ^l^ |  |  |  |  |  |  |
| Medscape ^m^ |  |  |  |  |  |  |
| Micromedex ^n^ |  |  |  |  |  |  |

^h^ Google is a free internet search software (www.google.com.br).

^i^ Google *Scholar* is a Google search engine for articles (https://scholar.google.com.br).

^j^ Scopus is an abstracts and citations database of peer-reviewed literature: scientific journals and books (https://www.scopus.com).

^k^ UpToDate is software that has technical information, access to drug monographs (Lexi-comp) and information on drug interactions. Restricted access to subscribers.

^l^ MD Consult is a portal for access to technical information and books. Access is restricted to subscribers (http://home.mdconsult.com)

^m^ Medscape is a portal to access technical information (with applications for Android and IOS), about medicines and diseases. Free access (http://portugues.medscape.com).

^n^ Micromedex is a drug information database with restricted access to subscribers. In Brazil, it is available in all university hospitals and to all Brazilian health professionals.

**11. Rate your ability to use the tools below:**

|  | Very Capable | Capable | Somewhat incapable | Incapable |
| --- | --- | --- | --- | --- |
| Google |  |  |  |  |
| Google schorlar |  |  |  |  |
| Bireme |  |  |  |  |
| PubMed |  |  |  |  |
| Scopus |  |  |  |  |
| UpToDate |  |  |  |  |
| MD Consult |  |  |  |  |
| Medscape |  |  |  |  |
| Micromedex |  |  |  |  |

**12. Please answer the following with your level of agreement**

|  | I totally agree | Partially agree | I do not agree or disagree | Partially disagree | Totally disagree |
| --- | --- | --- | --- | --- | --- |
| I have access to computer use in my work environment |  |  |  |  |  |
| I have internet access in my work environment |  |  |  |  |  |
| I have access to databases like UpToDate, MD Consult or other in my work environment |  |  |  |  |  |
| I need to expand my knowledge about using evidence-based information search tools to improve my performance in clinical activities |  |  |  |  |  |
| Having internet access helps in the pharmacist's clinical practice |  |  |  |  |  |

**13. In my workplace I use the computer to:**

Control and dispense medicines Exchange information by e-mail or intranet

 Search for technical information Other: _________________________

**14. The performance of YOUR clinical activities is hampered by:**

|  | I totally agree | Partially agree | I do not agree or disagree | Partially disagree | Totally disagree |
| --- | --- | --- | --- | --- | --- |
| Lack of priority by the management of the institutions about the importance of clinical pharmacy |  |  |  |  |  |
| Simultaneous clinical and non-clinical tasks |  |  |  |  |  |
| Lack of standardization of instruments for clinical pharmacy in Brazil |  |  |  |  |  |
| Low levels of knowledge of the practice of clinical pharmacy |  |  |  |  |  |
| Absence of electronic systems for documenting clinical pharmacy practice |  |  |  |  |  |

**15. How many hours per week do you dedicate EXCLUSIVELY to the practice of Clinical Pharmacy?**

 <2h

 2 - 6h

 7 - 12 h 13 - 24h

 25 - 40h

 I do not practice clinical pharmacy

**16. For how long have you been practicing Clinical Pharmacy in a hospital?**

 < 1 year

 1 - 5 years

 6 - 10 years

 11 - 20 years

 > 20 years

**17.** **In which type of health establishment do you practice Clinical Pharmacy?**

Public or Private:

 Public  Private

Place of establishment:

 Capital  Interior;

Size of establishment:

 Up to 50 beds;

 Between 51 to 150 beds

 >150 beds

**18. What practical activities of Clinical Pharmacy do you perform ?**

 Medicines Reconciliation

 In-hospital multidisciplinary visit

 Multidisciplinary home visit

 Daily analysis of prescriptions

 Drug-drug interaction analysis

 Drug-Food Interaction Analysis

 Guidance on dilution and drug stability for nursing staff

 Guidance on dilution and drug stability for medical staff

 Assessment of prescribed maximum and minimum doses

 Analysis of drug returns

 Pharmacovigilance

 Notification of adverse events

 Recording pharmaceutical recommendations in medical records

 Pharmaceutical recommendations to care team members

 Guidance on patient discharge

 Elaboration of transfer notes, with information about pharmaceutical care provided and sending with the patient at the time of transfer between units of the same hospital

 Elaboration of transfer notes, with information about pharmaceutical care provided and sending with the patient at the moment of their transfer between different hospitals

 Record my actions in the practice of the clinical pharmacy in printed form

 Record my actions in the practice of the clinical pharmacy in computerized system using fixed computer

 Record my actions in the practice of the clinical pharmacy in computerized system using tablet

 Other_________________

**19. What is the NAME of the software, for documenting clinical practice, that YOU USE?** Enter in the “Other” space the name of the computerized system, in which you record the actions of the practice of the clinical pharmacy in a hospital setting, if different from the options presented.

 I do not use software to record my practice. I register in paper form.

 Record my clinical practice in a word®-file.

 Record my clinical practice on a spreadsheet in Excel®.

 Register my clinical practice on a worksheet in Access®.

 Other: _____________________

**20. Is the software used to document your practice from the institution where you work?** They are considered as belonging to the hospital, when the software is available in the computers and tablets of the institution, and are used by the pharmacist**.**

 I do not use software for registration of actions in clinical pharmacy

 Yes, the software is from the hospital

 No. I use software from my particular computer / tablet.

**21. The computerized system for recording the clinical practice that you use IS FREE ACCESS?** Free access, should be understood, as one that does not require payment by the institution or by you, for installation, update and use.

 I do not use computerized system for registration of my actions in clinical pharmacy

 Yes  No

**22. When you go to publish or document actions and prepare reports of clinical pharmacy activities you:**

 Prepare spreadsheets in Excel

 Use directly (without typing or formatting) the reports issued by the hospital software

 Enter the data in statistical analysis software: SPSS

 Enter data in statistical analysis software: Epi Info

 Prepare tables in Word

 I do not report my clinical pharmacy actions

 Other: ______________

**23. For how long is the physical file of the clinical pharmacy activities kept by you or your hospital?** The physical files are the paper forms used in the practice of the clinical pharmacy. Documents and forms produced on paper during clinical pharmacy practice may be discarded or filed by the pharmacist for further consultation

I do not maintain physical file

Up to 1 year

1|- 2 years

2|- 5 years

5|- 10 years

≥10 years

**24. Would you use an electronic pharmacy practice registry system available on the internet?**

Yes No

**25. On the Features of a Clinical Pharmacy software, indicate your degree of agreement with the statements:**

|  | I totally agree | Partially agree | I do not agree or disagree | Partially disagree | Totally disagree |
| --- | --- | --- | --- | --- | --- |
| A clinical pharmacy software should be on the web, being easily accessible anywhere that has internet |  |  |  |  |  |
| A clinical pharmacy software should be easy |  |  |  |  |  |
| Clinical pharmacy software should securely archive information |  |  |  |  |  |
| Clinical pharmacy software should use standardized descriptors for archiving information |  |  |  |  |  |
| A clinical pharmacy software should display information in charts and tables |  |  |  |  |  |
| A clinical pharmacy software should look pleasing to the user |  |  |  |  |  |
| Clinical pharmacy software should perform descriptive statistical analyzes of data (mean, median, mode) |  |  |  |  |  |
| Clinical pharmacy software should be able to be used on tablet |  |  |  |  |  |
| Clinical pharmacy software should be able to be used on smartphones |  |  |  |  |  |

**26. Point out which functions, reports, and forms should a clinical pharmacy software to have in order to fully serve you: ___________________________**
